# Pathways and Roadblocks in Navigating Online Cancer Communities: Qualitative Study Among Young Adult Cancer Survivors

**DOI:** 10.2196/79893

**Published:** 2026-01-12

**Authors:** Qi Chen, Erin Donovan, Leyi Zhou, Lailea Noel, Barbara Jones

**Affiliations:** 1 Silberman School of Social Work, Hunter College, City University of New York, 2180 3rd Ave, Room 453, New York, NY, 10035, United States, 1 (212) 396-7586; 2 Department of Communication Studies, Moody College of Communication, The University of Texas at Austin, Austin, TX, United States; 3 School of Social Welfare, University of California, Berkeley, Berkeley, CA, United States; 4 Steve Hicks School of Social Work, The University of Texas at Austin, Austin, TX, United States; 5 School of Social Work, Boston University, Boston, MA, United States

**Keywords:** young adult, cancer survivors, online social networking, social support, health disparities

## Abstract

**Background:**

Online cancer communities provide young adult (YA) cancer survivors with access to informational and emotional support that may not be available in traditional care settings. While these platforms offer vital connection opportunities, the unique pathways YA survivors take to find online communities and the challenges they encounter remain underexplored.

**Objectives:**

This study aimed to (1) examine how YA cancer survivors locate and access online cancer communities and (2) identify barriers that impede their participation or sustained engagement.

**Methods:**

The first author conducted semistructured interviews with 12 YA cancer survivors aged 18 to 39 years who had experience using online resources after their diagnosis. Participants were recruited through purposive and snowball sampling from YA cancer-focused nonprofit organizations and through social media. Interviews were conducted via Zoom and analyzed using thematic analysis. The analytic process followed Braun and Clarke’s 6-phase framework and was supported by MAXQDA software. Codes and themes were generated inductively and refined iteratively.

**Results:**

Participants ranged in age from 24 to 39 years (mean 32, SD 5.08 years), with an average of 3 (SD 2.98) years since diagnosis. Most identified as female (n=9, 75%) and non-Hispanic White (n=7, 58%). Over half held a graduate degree (n=7, 58%), half were employed full time (n=6, 50%), and most resided in suburban areas (n=8, 67%). Cancer diagnoses included leukemia (n=3, 25%), lymphoma (n=4, 33%), and other solid tumors such as testicular, colon, and uterine cancers. At the time of the interview, 3 (25%) participants were in active treatment and 9 (75%) had completed treatment. Participants described five primary pathways to discovering online cancer communities: (1) direct searching using hashtags or keywords, (2) community hubs on public accounts, (3) referrals from health providers or social networks, (4) algorithm-recommended content, and (5) connections formed within preexisting online interest-based groups. Despite the promise of digital tools, participants encountered five roadblocks: (1) platform fragmentation and digital literacy complicated initial discovery; (2) lack of representation made it difficult for some to find communities where they felt seen; (3) emotional overload and engagement fatigue, along with shifting group hierarchies and boundaries, further hindered sustained participation; and (5) lastly, concerns about cyberbullying discouraged open engagement, prompting some to withdraw or limit their presence in online communities.

**Conclusions:**

YA cancer survivors navigated a fragmented and emotionally complex digital landscape in search of social support. Their ability to access and engage with online communities was shaped not only by individual agency and digital literacy but also by structural and relational factors. This study underscores the need for more discoverable, inclusive, and sustainable digital support environments. Oncology professionals and peer survivors can play a vital role in facilitating safe, informed access to online cancer communities. Multilevel psychoeducation and training with health care providers, YA cancer survivors, and online community facilitators are warranted to bridge gaps and enhance equity in digital survivorship care.

## Introduction

Before the widespread adoption of communication technologies, patients having cancer and their families largely relied exclusively on oncology providers for support and information. The rise of social media and online platforms has transformed this landscape, enabling cancer survivors to connect, share experiences, and find vital health information from the comfort of their homes. A report by the Pew Research Center revealed that 11% of adults in the United States have been following their friends’ health updates on online communities, and 5% have actively engaged by sharing their own health-related information, questions, or comments [1]. A national survey showed that the general population’s online support group participation grew from 5% in 2008 to 9% in 2017 [2].

Online cancer communities refer to groups of people with expressed interest in cancer who communicate using a website, social media platform, instant messaging, or app [3]. These communities, often created by patients, nonprofits, or health communication professionals, serve as supportive spaces for patients, survivors, and caregivers to seek information and emotional connection. Over time, members contribute to an evolving body of shared knowledge, fostering a sense of belonging based on common diagnoses or life circumstances. Cancer survivors report positive attitudes toward these online communities and the social support they provided [4].

Young adult (YA) cancer survivors, a distinct group of cancer survivors defined by the National Cancer Institute [5], often face unique challenges: social and developmental disruptions, feelings of isolation, and limited access to in-person support groups tailored to their needs [6]. Because YAs are often geographically dispersed, online cancer communities provide accessible spaces to exchange health information, share experiences, and find emotional and informational support among peers who understand their situations [7-9]. These communities foster empowerment as both a process and an outcome of participation, complementing traditional health care by promoting agency, peer learning, and self-management [10-12]. Participation can help survivors reclaim a sense of connection, reduce isolation, and build hope for life beyond cancer [13,14].

However, these benefits are not experienced uniformly. Engagement in online cancer communities is shaped by both individual characteristics, such as education level, health literacy, psychological well-being, and personality traits, and community-level factors like dynamics, including reciprocity, inclusiveness, and the quality of moderation [9,15,16]. Understanding how these factors interact to shape YA survivors’ participation remains critical to improving access to meaningful online support.

Finding an online community that meets one’s emotional and informational needs can be challenging for YA cancer survivors. Although there are various types of online cancer communities, 2 broad classifications, open forums and secret groups, pose distinct challenges for YA cancer survivors seeking meaningful support [17]. Open forums, typically hosted by charities or nonprofits, provide accessible and reliable information but foster limited personal connection, while secret groups, often patient-initiated and invitation-only, offer greater intimacy and emotional support but raise privacy concerns and require trust-building [17]. The diversity of platforms and fragmented resources can make exploration overwhelming. Privacy concerns are also particularly pressing for YAs, who fear being identified by future employers or wider social circles, making them gravitate toward more private groups that require referral to join [18]. However, the intimacy of secret groups comes at the cost of additional risks, including loss of control over shared stories [19,20], emotional distress following other members’ death [13,21], and exposure to misinformation or harmful content [22,23].

Taken together, these findings highlight the need to better understand how YA cancer survivors identify and engage with online health communities that align with their emotional and informational needs. Although prior studies have documented both the benefits and challenges of these spaces, little is known about how YAs navigate online resources, find suitable communities, and sustain meaningful participation. The purpose of this qualitative study is to understand (1) how YA cancer survivors locate and access their online cancer communities and (2) what hinders their ongoing engagement.

## Methods

### Design

This qualitative study used semistructured interviews to explore how YA cancer survivors find, access, and engage with online cancer communities. The study aimed to understand both the pathways through which participants discovered these communities and the barriers they encountered in participating or sustaining engagement over time.

### Participants

Purposive and snowball sampling was used to recruit participants. To be eligible for the study, participants needed to (1) receive a diagnosis of cancer at the age between 18 and 39 years, (2) be between 18 and 39 years at the time of the interview, (3) have experiences using social media platforms, and (4) be able to participate in a videoconference interview. The age range was based on the National Cancer Institute’s definition of adolescent and young adult (AYA) cancer survivors [5] and was selected to capture the relational and developmental contexts of young adulthood, a life stage marked by key social transitions and identity formation that shape survivorship experiences [6].

The research team developed a flyer that they asked nonprofit organizations dedicated to YA cancer to post on their social media accounts. Cactus Society and Livestrong Cancer Institute, 2 US-based organizations supporting AYA cancer survivors, shared the flyer on their social media accounts. The study flyer contained study aims, the principal investigator’s contact information, and a QR code of intake survey on Qualtrics. The principal investigator (QC) followed up with each potential participant to schedule Zoom interviews and gather verbal informed consent.

### Data Collection

All interviews followed the same interview guide with some variations based upon the flow of conversation between the participant and interviewer (see Multimedia Appendix 1 for the interview guide, including prompts and questions). The interview guide was developed by the research team. It contained questions related to the ways YA cancer survivors connected to peer survivors on social media, including positive and negative experiences during the process. The interviews were conducted in a one-on-one setting, free from the presence of any nonparticipants. Prior to each interview, participants were instructed to join from a private location and to ensure that no nonparticipants were present or able to overhear the conversation. Interviews were video recorded and transcribed by Zoom. Transcripts were made available for participant review upon request. The interviewer (QC) read through transcripts to ensure the quality of transcription and kept field notes that captured the main points of conversation after each interview.

### Data Analysis

Reflexive thematic analysis is a method for identifying, analyzing, and reporting patterns within data, defined by Braun and Clarke [24]. All the transcripts were deidentified before importing into MAXQDA (VERBI Software, 2021), a qualitative software program for data coding and comparison.

Two members of the research team, QC and LZ, independently coded all transcripts using an inductive, consensus-driven approach. The data coding approach was inductive. Codes and theme development were directed by the content of the data, rather than being theoretically driven [24]. Both coders initially reviewed a subset of transcripts to develop a preliminary codebook, which was refined through iterative discussion. Each transcript was then coded separately by both coders, followed by comparison and discussion to reconcile differences and reach agreement on code application and interpretation. Discrepancies were resolved through dialogue until full consensus was achieved, ensuring analytic consistency and rigor.

Following the six-phase process of thematic analysis, QC and LZ began with a process of (1) *data familiarization*, in which QC noted initial analytic observations about each data item and the entire dataset by reading the transcription and field notes. Two transcriptions were coded to (2) *generate initial codes* and develop the first-version codebook with columns of code category, code name, definition, eligibility, and example based on the initial codes. QC and LZ coded the other 2 interviews and revised the codebook accordingly by adding new codes, refining code’s eligibility criteria, and adding second-level codes to group the existing codes. After the codebook was finalized with all 12 interviews, the data were organized into a matrix with cases and second-level codes. QC and LZ (3) *searched for themes* by reading and identifying the similarity in data across cases in the matrix. The 2 authors (4) *reviewed the themes* and gave (5) *definitions and names to the themes*. All authors reached consensus regarding the themes’ consistency. The last phase was to move from semantic description to interpret the significance of patterns and their broader meaning and implications for the (6) final report.

### Rigor

Steps to ensure rigor were based on the trustworthiness criteria of data analysis posited by Lincoln and Guba [25]. The criteria involved establishing credibility, dependability, confirmability, and transferability. Throughout the research process, the first author reported the coding process and reflection of the transcription with the research team to assure the credibility of the analysis. The research team provided consultation and guidance on the naming of codes and themes to ensure the accuracy of language. The first author kept an audit trail (Koch [26]) to record each decision made with modifying the research question, finalizing the data analytic approach, and coding and analyzing the pattern to improve the dependability of the finding. Qualitative software was used, as it permits greater audibility and hence increases the reliability of the data analysis process.

Confirmability was sought by regular review and discussion of the codes with the research team by discussing reflexivity and how the interactive nature of the interview process influenced data collection. Member checking was not conducted, as this study employed a reflexive thematic analysis approach in which meaning is understood as coconstructed through researcher interpretation rather than verified by participants. Following Braun and Clarke’s distinction, traditional member checking assumes the existence of a single, verifiable truth and seeks to “correct” researcher subjectivity—an assumption that sits conceptually at odds with reflexive thematic analysis, where researcher subjectivity is viewed as an analytic resource, not a bias to eliminate [27]. Consistent with the interpretivist assumptions of reflexive thematic analysis, data saturation was not used as a criterion for determining sample adequacy because Braun and Clarke [28] advocated for achieving meaning through researcher interpretation.

### Ethical Considerations

Institutional review board approval was granted for this study by the University of Texas at Austin (protocol STUDY00003004). All study procedures complied with institutional and national research ethics standards for research involving human participants. Participants were informed of the study purpose, confidentiality protections, the benefits and risks of participation, and their rights to withdraw at any time. Prior to participation, all participants provided verbal informed consent to take part in the study and to be video recorded. Participation was voluntary, and participants were reminded that they could decline to answer any question or withdraw from the study at any time without penalty. All data were deidentified during transcription and analysis. Identifying information such as names or specific personal details was removed or replaced with pseudonyms. Digital recordings and transcripts were stored in a secure and access-restricted folder on UT Box, accessible only to the research team. Each participant received a US $20 Amazon electronic gift card, sent via email upon completion of the interview, as compensation for their time and contribution to the study.

### Positionality

The research team represents a diverse group of scholars and practitioners whose social identities, disciplinary backgrounds, and professional experiences shaped the design and interpretation of this study. QC (Asian woman, Assistant Professor of Social Work, and a PhD candidate at the time of the study), ED (White woman, Professor of Communication), LN (Black woman, Assistant Professor of Social Work), and BJ (White woman, Professor of Social Work) are seasoned qualitative researchers with a PhD degree and extensive experience studying psychosocial oncology, communication, and health equity among AYA cancer survivors. LZ (Asian woman, social work clinician, and doctoral student), the second coder of the transcript, brought both clinical practice experience and methodological expertise in qualitative research. The interviewer QC’s interest in this topic stems from her professional experience in psychosocial oncology and her commitment to addressing disparities in access to cancer support as well as the rising use and impact of communicative technology during the COVID-19 pandemic. She acknowledges that her background as a social work researcher and her own positive experiences with online communities as an immigrant may have shaped assumptions about online support and digital disparities. As someone who is not a cancer survivor herself, she remains mindful of the potential distance between her professional perspective and participants’ lived experiences. QC had limited prior relationships with participants. One participant was a personal friend of QC, while all others had no prior relationship with the interviewer. Participants were informed about QC and the study through the introductory phone calls or emails and the consent form, which described QC’s role as the principal investigator and explained the study’s purpose, goals, and procedures. No additional personal or professional information was shared beyond what was stated in the consent materials.

## Results

### Participants’ Characteristics

**Figure 1.**
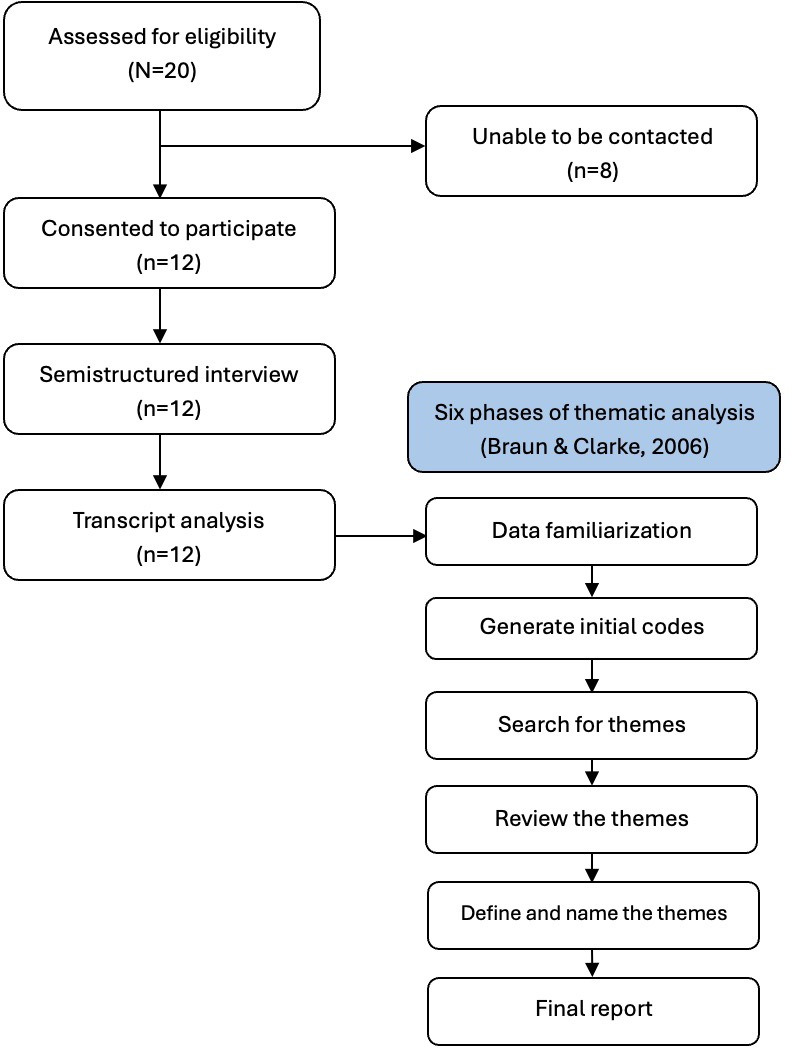
Participant recruitment and thematic analysis process flowchart [24].

A total of 20 potential YA participants who filled out the screening questionnaire were all eligible for the study. Of these 20 YAs, 8 did not respond to the principal investigator’s emails or phone calls, and 12 consented and were recruited to participate in the interviews, yielding an enrollment rate of 60%. Figure 1 presents the recruitment and analysis process flowchart. Semistructured individual Zoom interview data were collected from September 2022 to February 2023, ranging from 39 to 110 minutes (mean 67.2 min, SD 21.3 min). No repeated interview was carried out, and no participants were requested to review or edit their transcripts. The demographic information of participants is presented in Table 1. Participants had a mean age of 32 (SD 5.08; range 24‐39) years and an average of 3 (SD 2.98; range 0‐11) years since diagnosis. Most participants were identified as female (n=9, 75%). The majority were non-Hispanic White (n=7, 58%), followed by Hispanic (n=2, 17%) and Asian (n=2, 17%), and 1 (8%) identified as multiracial. Over half (n=7, 58%) of participants held a graduate degree, while 4 (33%) had completed some college or a college degree, and 1 (8%) had completed high school. In terms of employment, 6 (50%) participants were employed full-time, 2 (17%) part-time, and 4 (33%) not employed. Participants resided in 8 different states. Most participants resided in suburban areas (n=8, 67%). Cancer diagnoses among participants varied, with leukemia being the most common (n=3, 25%), followed by Hodgkin’s lymphoma (n=2, 17%), testicular cancer (n=2, 17%), and individual cases of non-Hodgkin’s lymphoma, colon, brain, soft tissue, and uterine cancers (each n=1, 8%). At the time of the interview, 9 (75%) participants had completed treatment, while 3 (25%) were still in active treatment.

**Table 1.** Participant demographics and background information (n=12).

Demographics	Count
Age, mean (SD; range)	32 (5.08; 24-39)
Years since diagnosis, mean (SD; range)	3 (2.98; 0‐11)
Sex, n (%)	
Female	9 (75)
Male	3 (25)
Race, n (%)	
Non-Hispanic White	7 (58.3)
Hispanic	2 (16.7)
Asian	2 (16.7)
Multiracial	1 (8.3)
Education attainment, n (%)	
High school	1 (8.3)
Some college or college	4 (33.4)
Graduate school	7 (58.3)
Employment, n (%)	
Full time	6 (50)
Part time	2 (16.7)
Not employed	4 (33.3)
Location (State), n (%)	
Texas	3 (25)
New York	2 (16.7)
Illinois	2 (16.7)
Pennsylvania	1 (8.3)
Wisconsin	1 (8.3)
Florida	1 (8.3)
Maryland	1 (8.3)
Michigan	1 (8.3)
Urbanicity, n (%)	
Urban	4 (33.3)
Suburban	8 (66.7)
Cancer diagnosis, n (%)	
Leukemia	3 (25)
Hodgkin’s Lymphoma	2 (16.7)
Testicular cancer	2 (16.7)
Non-Hodgkin’s Lymphoma	1 (8.3)
Colon cancer	1 (8.3)
Brain cancer	1 (8.3)
Soft tissue cancer	1 (8.3)
Uterine cancer	1 (8.3)
Treatment status, n (%)	
Completed	9 (75)
In active treatment	3 (25)

### Qualitative Findings

This study explored 2 central questions: (1) how YA cancer survivors locate and access online cancer communities and (2) what challenges they encounter in sustaining engagement. The findings revealed a range of entry points that survivors used to discover supportive online spaces, as well as structural, interpersonal, and emotional factors that limited their continued participation. While participants represented a range of ages within the YA category (24‐39 y), their approaches to engaging with online cancer communities and the obstacles they experienced were broadly similar. No age-related patterns were observed in terms of search strategies, preferred platforms, or engagement levels.

**Figure 2.**
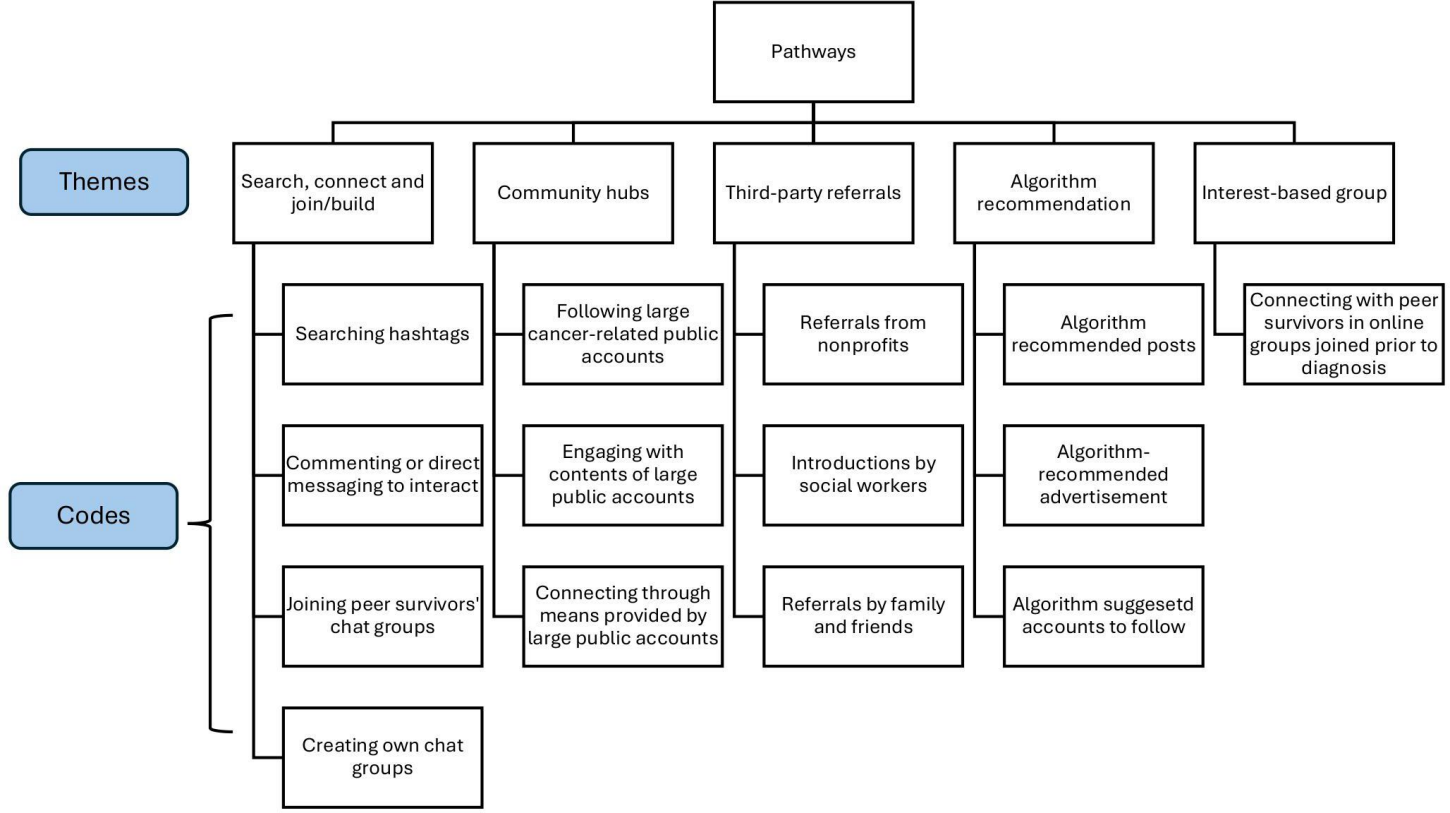
Coding tree of “Pathways” illustrating how young adult cancer survivors discovered online cancer communities.

The results are presented in 2 sections, corresponding to each research question. The first section, *Pathways*, included 5 themes describing how YA cancer survivors found online communities: (1) search, connect, and join/build, (2) community hubs, (3) third-party referrals, (4) algorithm recommendations, and (5) online interest-based groups. The second section, *Roadblocks*, outlined 5 themes that capture barriers to sustained engagement: (1) platform fragmentation and digital literacy, (2) group boundaries and hierarchies, (3) lack of representation, (4) emotional overload and engagement fatigue, and (5) cyberbullying. Figures2  3 depicted 2 coding trees of Pathways and Roadblocks to illustrate the codes identified and themes emerged in the analysis. Table 2 presents each theme along with its definition and representative participant quotes.

**Figure 3.**
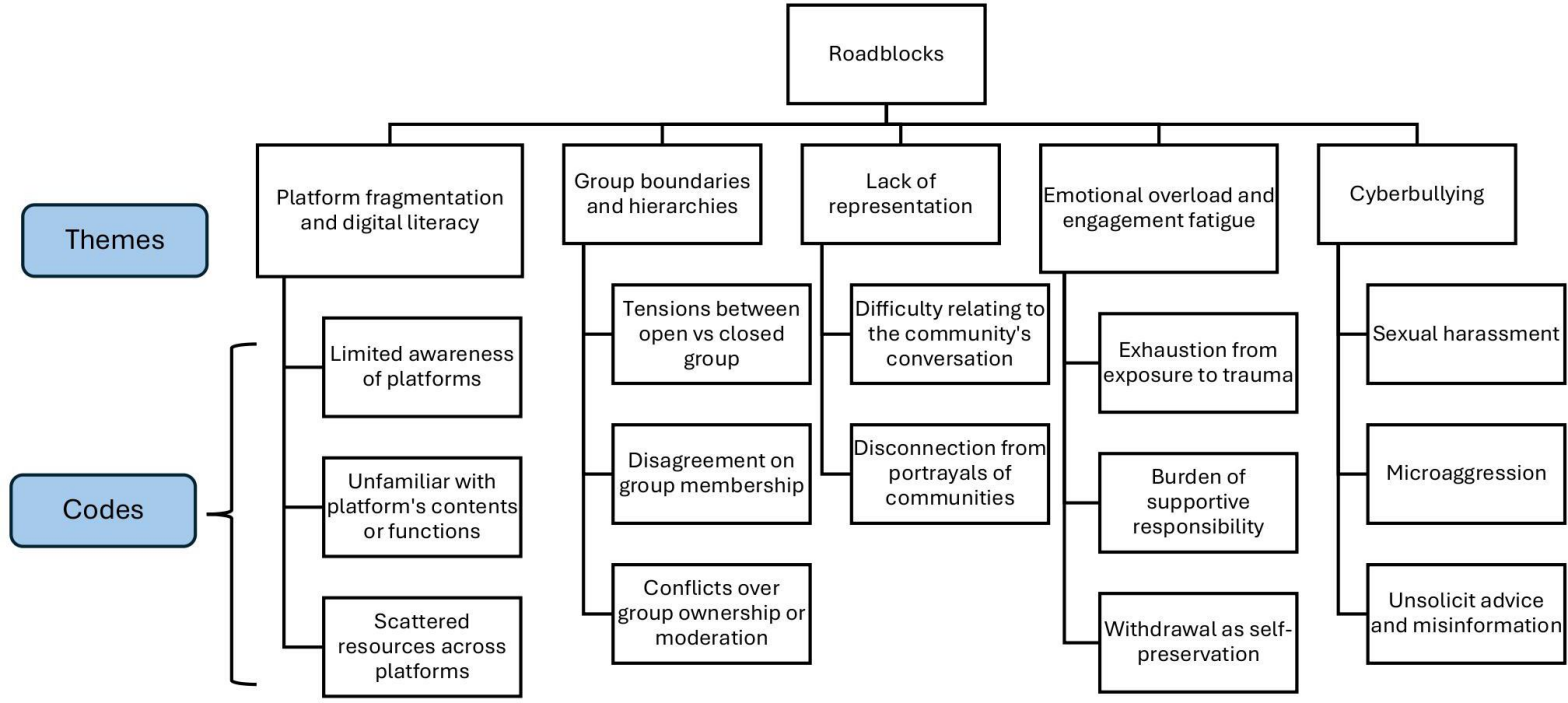
Coding tree of “Roadblocks” illustrating barriers that young adult cancer survivors encountered in sustaining engagement with online cancer communities.

**Table 2.** Themes, description, and representative quotes: pathways and roadblocks.

Themes	Description	Representative quote
Pathways		
Search, connect, and join/build	YA survivors actively searched relevant hashtags and initiated contact with peer survivors through comments or direct messages, leading to joining an existing community or forming their own community	*She has a decent following, so I didn’t expect her to see it or anything, but she responded back. So I messaged her, and then suddenly started messaging and then exchanged phone numbers and she’s like, I’ve met a few other girls, who also going through cancer treatment this summer. So then she made a group chat for us. And then we said we all will support each other through cancer treatment this summer, when we should like having fun or whatever. That was like eight months ago, we still talk almost like every day.* (Participant 09) *First of all, I was adding people that were in my state to try and build a community in my state, then it widened out more and more. And we would eventually share each other’s Instagrams and that’s how I built it up. Sometimes I’ll still search up care packages, just to see if there’s any new ones to add to my list, but also to see any people out there so I can connect with them.* (Participant 10)
Community hubs	YA cancer survivors followed large public accounts serving as information hubs for peer interaction.	*The person who runs the account made a spreadsheet where people could put their name, their age, their diagnosis, and where they lived and the Instagram handle. And then, if anybody wanted to talk to them they could reach out. So I put my information on that spreadsheet, and I had one person reach out*. (Participant 08)
Third-party referrals	Nonprofit organizations or health care providers referred YA cancer survivors to existing online groups.	*The other organization does a weekly happy hour every Friday, and that is the same host every time, and I started going and I found out from people who go to that also go to Stupid Cancers zoom session. And so, there’s a lot of cross involvement with different survivors are part of and take part in programming for different AYA groups. Some of my closest friends that I’ve connected with there, I have almost all of them on Instagram and/or Facebook. I talk with them on an Instagram group Chat* (Participant 06).
Algorithm recommendations	YA cancer survivors encountered groups through social media algorithm suggestions.	*When I search for something on Google, Facebook picks up my information and pushes related content… I can even see on Facebook what I had just searched for on Google.* (Participant 01) *That was through algorithm. I didn’t search for anything during that time. I think I just got lucky that one day, and she already knew people. That was like pure luck, like it was meant to happen. I don’t think I find that many people on social media share similar experiences*. (Participant 09)
Online interest-based groups	YA cancer survivors found peer support within preexisting online interest or hobby groups.	*It wasn’t a cancer group, but people there had similar ages and life experiences, so I felt connected.* (Participant 07)
Roadblocks		
Platform fragmentation and digital literacy	YA cancer survivors struggled to locate reliable supportive communities due to scattered platforms and lack of digital literacy.	*I didn’t really use Facebook and Instagram much so I was not aware of those accounts. I used to only use Chinese-language platforms and those that are available for Chinese people. I like hanging out with other Chinese people more*. (Participant 01)
Group boundaries and hierarchies	Conflicts over group ownership, membership criteria, or tone led to disengagement.	*Sometimes caregivers come in, and they’ll tell us ‘Oh, how inspirational you are.’ And we just don’t know how to take that because we’re like, we’re just trying to live our life or don’t feel inspirational*. (Participant 10) *It fell apart because there was a disagreement over who was old enough to be in the group, and that hurt some people’s feelings… and another group (that was dismissed), it turned out one person was making stuff up and people just didn’t want to be a part of the group*. (Participant 06)
Lack of representation	Limited diversity (age, diagnosis, culture) left survivors feeling disconnected or unseen.	*I couldn’t really find a relevant account about infertility that felt like it described my experience. I followed some infertility-related accounts that weren’t cancer specific, but just about people who had infertility in general, and those accounts are mostly run by white women who are really religious and really Christian. And so I think I’ve decided not to engage with those accounts anymore, just because I couldn’t find anybody who had experiences that were similar to me or identities that were similar to mine*. (Participant 08)
Emotional overload and engagement fatigue	Exposure to others’ trauma and constant emotional demands led to burnout and withdrawal.	*I already felt miserable and no one cared about me... but seeing them vent still made me feel awful… On top of my own problems, I have to carry others’ misery… thinking about their fate makes it unbearable.* (Participant 01) *Sometimes it can feel a little repetitive. Like there are certain types of posts that I feel like you see again and again and again. So I think that can make it hard to stay engaged.* (Participant 03)
Cyberbullying	Harassment or microaggressions led survivors to privatize accounts, reducing visibility and engagement.	*There’s a subsection of people who are really weird that like bold girls. They will message cancer patients with really graphic descriptions. I’ve got that kind of message*. (Participant 10)

### Pathways

YA cancer survivors accessed online communities through a range of entry points that reflected both deliberate strategies and serendipitous discovery.

#### Search, Connect, and Join/Build

This was the most common way YA cancer survivors mentioned regarding finding and joining their online cancer community. Usually, it would start with searching for keywords related to their cancer diagnosis or type of treatment, or the hashtag #AYAcancer, #YoungAdultCancer, #AYAcancer, or #cancerinyour20s, to identify peer YA survivors who shared similar cancer experiences. Some YA cancer survivors would initiate the conversation by commenting on the post of accounts they identified, such as sending wishes and making comments on similar cancer-related situations they share, and some would directly message to connect. After the connection was established and a larger network based on similar cancer experiences was formed, sometimes a chat group/channel would be created by one of the group members. There were also some other YA cancer survivors taking a leading role in creating a community for people they knew and actively searching out to YA cancer survivors who might be looking for support on social media.

#### Community Hubs

Community hubs were centralized online spaces managed by individuals or small groups, serving as anchors or gatekeepers for connection, information exchange, and peer support. We found that there were such hubs that existed for YA cancer survivors to connect: almost all of the participants followed the accounts @thecancerpatient and @NoGriefTouristAllowed on Instagram. These accounts shared similar traits: large number of followers and mostly YA cancer survivors. They served as an information hub for YA cancer survivors. A YA cancer survivor community was naturally formed based on these 2 accounts, and these accounts were acknowledged and advertised by these cancer survivors at platforms other than Instagram. These public accounts connected peer survivors through posts that reflected shared cancer experiences. People responded in the comments or initiated conversations or through “story” (posts visible for 24 h) featuring followers’ requests to connect. Some accounts would create ways to collect the contact information of those who want to connect.

#### Third-Party Referrals

YA cancer survivors also found nonprofit organizations or social workers at the hospital or cancer center helpful in referring to reliable online support resources. Nonprofit organizations dedicated to adolescent and YA cancer care, such as Stupid Cancer, Elephants and Tea, and Cactus Cancer Society, usually host online support groups and have their own forum or social media accounts. YA cancer survivors described being connected across different organizations, participating in multiple online cancer communities, and then forming their own communities in other social media platforms. YA cancer survivors also shared that when their family and friends found related resources, they would also recommend them and encourage them to connect. For instance, one participant mentioned that her sister has been an active user on the Reddit Cancer Caregiving subreddit and recommended her to join a cancer-related Reddit channel to find the community.

#### Algorithm Recommendation

As algorithm recommendation technology continued to advance, YA cancer survivors also benefited from recommendations on websites and apps they visited. Some described seeing the advertisement of Leukemia Society on Facebook after multiple rounds of internet search about Leukemia and survival rates and ended up visiting the site and found peer support programs. Some described their experiences of being recommended social media personal accounts with contents that were relevant to their life experiences and being able to connect with those people.

#### Online Interest-Based Groups

YA cancer survivors were comfortable with communication technologies, and they browsed different online communities at an early age. Therefore, many of them were already part of some online communities before they got cancer. One participant described her experience being in one huge interest group community, and there was a subgroup with people diagnosed with different types of cancer and stages of cancer. Even though it was not specifically for YAs, the participant described that since these interest-based groups were usually formed with people of similar age, demographic, and political outlook, or shared hobbies, it was easier to feel connected to these people.

### Roadblocks

While online communities offered vital spaces of support, YA cancer survivors reported a number of challenges that shaped their participation.

#### Platform Fragmentation and Digital Literacy

Participants described difficulties locating relevant support communities due to the fragmented nature of social media platforms. Information and resources were scattered across platforms such as Instagram, Facebook, Reddit, and organization-specific forums, many of which lacked centralized searchability. Some participants were unfamiliar with widely used platforms in the United States, particularly immigrant YA cancer survivors who previously used regional platforms to maintain relationships with family and friends back in their home countries. This limited exposure made it challenging to access content or locate established YA cancer communities on English social media platforms.

Others noted that even when they were familiar with a platform, they often lacked the knowledge of which accounts or groups to follow. Many were also unaware of nonsocial media–based online communities. For instance, one participant shared her experience of only recently learning about COLONTOWN, a web-based platform uniquely for colorectal cancer survivors. Furthermore, many shared that YAs who were connected through one platform might move to another to create their own group, sometimes choosing platforms others had never used or did not have accounts on. These access disparities underscored how platform fragmentation and varying levels of digital literacy could hinder survivors’ ability to locate and engage with appropriate sources of support.

#### Group Boundaries and Hierarchies

Online community boundaries could be challenging to manage. In open forums, lacking group boundaries would trigger uncomfortableness and conflicts and thus decrease their interests in staying in the group. Participants stated that for some groups that also welcomed the participation of caregivers, family members, or providers would interrupt the group dynamics and disrupt the sense of safety and belonging of the community. In secret/closed groups, group hierarchy, group ownership, and group membership could also become problematic. If agreement could not be achieved between group owner and group members, it would cause conflict, and the online group would have to be dismissed if conflicts continue to escalate.

#### Lack of Representation

Not all online cancer communities effectively met the informational and emotional support needs of YA cancer survivors. Several participants in the study described feeling like “outsiders” in these spaces. A major barrier to meaningful engagement was the perceived lack of inclusiveness and representation. Participants emphasized that the characteristics of community members, such as age, diagnosis, religion, health beliefs, cultural background, and treatment experience, strongly influenced their sense of connection. When these attributes did not align, it was difficult to relate to others or feel understood. For example, wide variations in treatment plans and side effects among YA patients having cancer made it challenging to find peers with similar experiences. The more disconnected participants felt from the community, the less likely they were to engage.

Some YA cancer survivors expressed discomfort with content on accounts that were community hubs and overly romanticized the cancer experience. They found such narratives to be superficial and disconnected from their own realities, making it harder to relate or feel represented. One participant shared that she avoided public accounts that presented highly curated or idealized portrayals of survivorship, as they felt inauthentic and failed to meet her informational or emotional needs.

#### Emotional Overload and Engagement Fatigue

For many YA cancer survivors, ongoing exposure to peers’ illness narratives in online communities could generate a deep sense of emotional fatigue, like one participant said: “Everyone’s unloading their trauma and it’s overwhelming.” (Participant 05). This exhaustion stemmed from not only the content’s intensity but also the complex dynamics of shared vulnerability and responsibilities. This form of emotional overload often coexisted with a sense of obligation to support, which heightened the burden. In addition to this emotional overload, participants described seeing similar posts over time could become draining. These revealed not a loss of interest but the emotional cost of showing up, where survivors must balance their need for support with the strain of witnessing others’ ongoing pain. Self-preservation, through withdrawal or silence, became a necessary response for survivors negotiating their own healing within communal spaces of vulnerability.

#### Cyberbullying

For people to be able to search one’s posts or follow one’s account, one needs to keep their account and posts public. However, being public and exposing themselves to the internet could be risky, especially for YA survivors. Many participants reported microaggression and cyberbullying on social media, such as unfriendly comments on their appearance, unsolicited advice, or even sexual harassment. As a result, YA cancer survivors who keep their accounts public to share their cancer experiences and help peer survivors connect initially would change their account setting to private to avoid these negative impacts. The unsafe environment on social media would limit the information source that peer YA cancer survivors could access.

## Discussion

### Overview

This study provided timely insights into how YA cancer survivors navigated the increasingly complex landscape of online cancer communities. It identified 5 key pathways to discover online communities and 5 major barriers in finding and sustaining engagement. The findings revealed that survivors’ participation was influenced not only by personal agency and constraints but also by systemic factors such as platform accessibility and usability. The findings also further illuminated understudied interpersonal dynamics, such as emotional labor and shifting hierarchies and boundaries within communities, that affected survivors’ sustained involvement in online communities. By centering YA cancer survivors’ lived experiences in exploring online communities, this research advanced a more nuanced understanding of how to foster inclusive, meaningful, and sustainable online support communities.

### Principal Findings

This study uncovers new forms of digital space navigation and structural inequity that shape survivors’ ability to benefit from online support. Our findings showed that with more online platforms available, YA survivors joined or formed communities across different platforms and often switched between them. While new features such as algorithmic recommendations, hashtags, stories, and group chat channels allowed them to find or form online cancer communities more easily, the benefits of these tools were not experienced equitably. Participants’ capacity to navigate and engage effectively was strongly influenced by their familiarity with functions of different platforms, many of which were not accessible to all users. For instance, immigrant survivors originally from countries where platforms like Facebook and Instagram are banned often lacked exposure to dominant US platforms. It demanded additional effort to learn and transition to a different platform solely for the purpose of accessing online support. Furthermore, limited access to certain social media platforms among users from specific cultural backgrounds results in fewer online support communities offering inclusive and culturally relevant content in their native languages. This disproportionately affected YA of color. These gaps in representation were compounded by algorithmic patterns that reinforced misinformation and unsolicited advice, disproportionately affecting those with lower health literacy or limited English proficiency. Together, these findings reveal how structural digital inequities shaped YA survivors’ capacity to benefit from online support.

This study reveals that YA survivors’ engagement with online cancer communities was emotionally dynamic and relationally fluid. Their interactions were marked with uncertainty and change. Navigating online cancer communities involved ongoing assessment of the relevance of the communities, negotiation of personal or group boundaries, and weighing between benefits and the risks. Some participants moved fluidly between communities or platforms as new communities emerged and migrated, while others withdrew temporarily or permanently when the emotional toll became too high. Rather than serving as a stable or consistently supportive resource, online cancer communities could also generate emotional distress, uncertainty, and exposure to unsafe or overwhelming virtual environments.

### Comparison to Prior Work

This study extends prior digital health disparity research on YA survivors’ online support by shifting attention from individual constraints in digital literacy to structural inequities in virtual spaces. Prior research has largely emphasized individual-level factors such as education, socioeconomic status, or their social capital as the main drivers of digital divide in access and utilization of online resources [29,30]. Our study reveals that digital disparities persisted even among digitally savvy YA survivors, driven by the nature of online platforms. Participants’ platform preferences were shaped by the habits as well as the accessibility of apps and websites of their families and social networks [30]. One prior study has similarly identified representation barriers, which noted that YA survivors often avoided older platforms such as Facebook, where most established cancer support groups exist, due to perceived misalignment with their peer networks and identity [13]. Familiarity with specific platforms, access to culturally relevant content, and the design of digital infrastructures emerged as key determinants equally impacted YA. This shift from individual to systemic explanations underscores the importance of improving platform accessibility and usability.

This study also advances existing literature by revealing how interpersonal dynamics within online communities could introduce more emotional distress and uncertainties besides support. Consistent with the prior findings, our study found that YA cancer survivors had strong needs of connecting to peer survivors [31] and they would search online proactively to find peer survivors [32]. Our study pointed out that, however, engagement was not always self-initiated; algorithms or others’ suggestions often prompted participation, leading to interpersonal strain. Prior work has well documented the benefits for YA cancer survivors involving online cancer communities, emphasizing information exchange, social support, and empowerment [31,33,34]. Risks such as exposure to distressing content or misinformation have also been acknowledged, though often discussed as isolated, individual experiences [35]. One prior study has examined the dynamics of social media engagement among YA cancer survivors, but focusing primarily on individual motivation and preferences [32]. Our findings expand these understandings by showing that group-level factors such as changing boundaries, evolving hierarchies, and the associated emotional labor directly influence engagement and withdrawal. By framing these experiences as relational and evolving, this study highlights that community organizing and group moderation is necessary for virtual spaces.

### Limitations

This study has several limitations. First, our sample included only participants who had internet access and prior experience using social media. It remained unclear whether YA cancer survivors without such access or experience would be open to, or able to engage with, online cancer communities. This inclusion criterion was necessary to ensure that participants could reflect meaningfully on their experiences navigating online communities. However, it may have limited the perspectives of YA cancer survivors with limited digital access or literacy or who lack interests in online cancer communities. Future studies should consider various recruitment strategies (eg, phone interviews or community-based outreach) to capture the experiences of survivors who have not been involved with any online cancer communities.

Second, the sample lacked representation from African American participants. Prior research indicated that African American cancer survivors were underrepresented in online support groups and less likely to use the internet for health information compared to their White counterparts, due in part to digital access barriers, differing help-seeking preferences, and lower trust in online health sources [36,37]. Although we recruited through multiple nonprofit networks serving diverse populations and encouraged participant referrals, our sample may have unintentionally excluded survivors not affiliated with such organizations or those relying on offline or culturally specific support systems. Future research should employ more inclusive recruitment strategies, including outreach through hospital registries, partnerships with African American survivor networks or community centers, and targeted social media campaigns or accounts that have higher number of African American followers. Addressing these structural and digital inequities is essential to advancing equity and representation in digital cancer survivorship research.

### Future Directions

Future intervention research and clinical practice should focus on improving the accessibility and sustainability of online cancer communities through both structural support and individual capacity-building.

At the system level, oncology social workers have long played a critical role in connecting patients with fragmented resources [38], and our findings further confirmed this essential function. However, many social workers, particularly those outside of large cancer centers, were not well-versed in the availability of online cancer communities and virtual support groups for minority YA survivors on various scattered platforms [31]. While social support from family, peers, and health care providers can facilitate access to online communities, the active involvement of health care professionals or trained moderators in online health communities enhances trust, reduces the spread of misinformation, and mitigates risks such as cyberbullying and communication breakdowns [39,40]. Expert-led moderation has been associated with improved emotional safety, greater engagement, and more supportive peer interactions [41]. This underscores the importance of digital literacy training with health care professionals to strengthen their competencies in navigating and moderating different online cancer communities, so they can more effectively refer, prepare, and support YA survivors.

At the individual level, private or peer-led groups typically rely on YA survivors themselves to establish norms and maintain boundaries, placing added emotional and relational burdens on those already coping with cancer. Our findings indicated that many YA survivors not only passively join a group but actively take part in building or contributing to their own communities. One promising approach is to train YA cancer survivors who are interested in leadership roles in online communities on skills to identify harmful content, establish group norms, and manage conflict. Additionally, while the prevalence of cyberbullying and emotional risk in online communities has been documented [42], few interventions exist to prepare survivors for these challenges. Education around safe storytelling, self-care, and managing online engagement fatigue should be integrated into survivorship support programming [35]. This includes guidance on when to disengage, how to set personal boundaries, and how to respond to toxic interactions.

### Conclusion

This study highlights that YA cancer survivors navigate online communities through multiple pathways but face significant barriers to sustained engagement, highlighting the need for provider training and peer moderator support. To foster safer and more inclusive online environments, health care providers should be trained to guide survivors toward reliable support resources; community moderators should be trained to manage emotional labor and maintain healthy group boundaries; and YA cancer survivors should receive survivorship support to navigate online spaces effectively. Strengthening these multilevel supports can enhance the sustainability and psychosocial benefits of online cancer communities for YAs.

## Supplementary material

10.2196/79893 Multimedia Appendix 1 Interview guide.

10.2196/79893 Checklist 1 COREQ checklist.

## Abbreviations

AYA: adolescent and young adult
YA: young adult

## References

[1] Fox S (2014). The social life of health information. https://www.pewresearch.org/short-reads/2014/01/15/the-social-life-of-health-information/.

[2] Chou WYS, Gaysynsky A, Trivedi N, Vanderpool RC (2021). Using social media for health: national data from HINTS 2019. J Health Commun.

[3] Gupta T, Schapira L (2018). Online communities as sources of peer support for people living with cancer: a Commentary. J Oncol Pract.

[4] Jansen F, van Uden-Kraan CF, van Zwieten V, Witte BI, Verdonck-de Leeuw IM (2015). Cancer survivors’ perceived need for supportive care and their attitude towards self-management and eHealth. Support Care Cancer.

[5] (2025). Adolescent & Young Adult Health Outcomes & Patient Experience Study (AYA HOPE). National Cancer Institute (NCI).

[6] Zebrack BJ, Block R, Hayes-Lattin B (2013). Psychosocial service use and unmet need among recently diagnosed adolescent and young adult cancer patients. Cancer.

[7] Sansom-Daly UM, Wakefield CE, Ellis SJ (2021). Online, group-based psychological support for adolescent and young adult cancer survivors: results from the recapture life randomized trial. Cancers (Basel).

[8] Viola A, Panigrahi G, Devine KA (2020). Digital interventions for adolescent and young adult cancer survivors. Curr Opin Support Palliat Care.

[9] Vlooswijk C, Janssen SHM, Sleeman SHE (2024). Identifying the informational needs and sources of support of adolescent and young adult (AYA) cancer survivors to inform the development of a digital platform. J Cancer Surviv.

[10] Johansson V, Islind AS, Lindroth T, Angenete E, Gellerstedt M (2021). Online communities as a driver for patient empowerment: systematic review. J Med Internet Res.

[11] Verberne S, Batenburg A, Sanders R, van Eenbergen M, Das E, Lambooij MS (2019). Analyzing empowerment processes among cancer patients in an online community: a text mining approach. JMIR Cancer.

[12] Frost J, Massagli M (2009). PatientsLikeMe the case for a data-centered patient community and how ALS patients use the community to inform treatment decisions and manage pulmonary health. Chron Respir Dis.

[13] Lazard AJ, Collins MKR, Hedrick A (2021). Using social media for peer-to-peer cancer support: interviews with young adults with cancer. JMIR Cancer.

[14] Daniels SR, Yang CC, Toohey SJ, Willard VW (2021). Perspectives on social media from adolescents and young adults with cancer. J Pediatr Oncol Nurs.

[15] Badreddine BM, Blount Y (2021). Understanding influential factors behind lurking behaviour in online cancer communities. Behav Inf Technol.

[16] Park KA, Eum SY, Oh H (2020). Factors affecting online health community participation behavior in patients with thyroid cancer. PLoS ONE.

[17] Harkin LJ, Beaver K, Dey P, Choong KA (2020). Secret groups and open forums: defining online support communities from the perspective of people affected by cancer. Digit Health.

[18] Clerici CA, Quarello P, Bergadano A (2018). Proper use of social media by health operators in the pediatric oncohematological setting: consensus statement from the Italian Pediatric Hematology and Oncology Association (AIEOP). Pediatr Blood Cancer.

[19] Easley J (2019). Motivations for cancer history disclosure among young adult cancer survivors. J Cancer Surviv.

[20] Pini S, Hugh-Jones S, Shearsmith L, Gardner P (2019). “What are you crying for? I don’t even know you” - the experiences of teenagers communicating with their peers when returning to school. Eur J Oncol Nurs.

[21] Harkin LJ, Beaver K, Dey P, Choong K (2017). Navigating cancer using online communities: a grounded theory of survivor and family experiences. J Cancer Surviv.

[22] DiFonzo N, Robinson NM, Suls JM, Rini C (2012). Rumors about cancer: content, sources, coping, transmission, and belief. J Health Commun.

[23] Hughes B, Joshi I, Wareham J (2008). Health 2.0 and Medicine 2.0: tensions and controversies in the field. J Med Internet Res.

[24] Braun V, Clarke V (2006). Using thematic analysis in psychology. Qual Res Psychol.

[25] Lincoln YS, Guba EG (1985). Naturalistic Inquiry.

[26] Koch T (1994). Establishing rigour in qualitative research: the decision trail. J Adv Nurs.

[27] Braun V, Clarke V (2023). Toward good practice in thematic analysis: avoiding common problems and be(com)ing a knowing researcher. Int J Transgend Health.

[28] Braun V, Clarke V (2021). To saturate or not to saturate? Questioning data saturation as a useful concept for thematic analysis and sample-size rationales. Qual Res Sport Exerc Health.

[29] Zhang Y, Xu P, Sun Q, Baral S, Xi L, Wang D (2023). Factors influencing the e-Health literacy in cancer patients: a systematic review. J Cancer Surviv.

[30] Mohsin FM, Ali SH, Chong SK, Parikh RS, DiClemente RJ, Hu L (2023). Social media utilization within Asian American families and its role in healthy lifestyle behavioral influence: results from a nationwide survey. Social Media + Society.

[31] Kent EE, Smith AW, Keegan THM (2013). Talking about cancer and meeting peer survivors: social information needs of adolescents and young adults diagnosed with cancer. J Adolesc Young Adult Oncol.

[32] Lazard AJ, Collins MKR, Hedrick A (2021). Initiation and changes in use of social media for peer support among young adult cancer patients and survivors. Psychooncology.

[33] Zebrack B (2008). Information and service needs for young adult cancer patients. Support Care Cancer.

[34] Lazard AJ, Meernik C, Collins MKR (2023). Social media use for cancer support among young adults with cancer. J Adolesc Young Adult Oncol.

[35] De Clercq E, Rost M, Gumy-Pause F, Diesch T, Espelli V, Elger BS (2020). Moving beyond the friend-foe myth: a scoping review of the use of social media in adolescent and young adult oncology. J Adolesc Young Adult Oncol.

[36] Fogel J, Ribisl KM, Morgan PD, Humphreys K, Lyons EJ (2008). Underrepresentation of African Americans in online cancer support groups. J Natl Med Assoc.

[37] Fareed N, Swoboda CM, Jonnalagadda P, Huerta TR (2021). Persistent digital divide in health-related internet use among cancer survivors: findings from the Health Information National Trends Survey, 2003-2018. J Cancer Surviv.

[38] Zebrack B, Kayser K, Padgett L (2016). Institutional capacity to provide psychosocial oncology support services: a report from the Association of Oncology Social Work. Cancer.

[39] Wadden D, August T, Li Q, Althoff T (2020). The effect of moderation on online mental health conversations. arXiv.

[40] Huh J, Marmor R, Jiang X (2016). Lessons learned for online health community moderator roles: a mixed-methods study of moderators resigning from WebMD communities. J Med Internet Res.

[41] Bizzotto N, de Bruijn GJ, Schulz PJ (2023). Buffering against exposure to mental health misinformation in online communities on Facebook: the interplay of depression literacy and expert moderation. BMC Public Health.

[42] Reuman H, Kerr K, Sidani J (2022). Living in an online world: social media experiences of adolescents and young adults with cancer. Pediatr Blood Cancer.

