# Supplementary material for: Pathways and Roadblocks in Navigating Online Cancer Communities: Qualitative Study Among Young Adult Cancer Survivors
Source: JMIR Cancer. 2026 Jan 12;12:e79893. doi: 10.2196/79893 (PMC12795408; doi:10.2196/79893)
Supplement: Multimedia Appendix 1 [file cancer-v12-e79893-s001.docx]

**Interview Guide**

Thank you for taking the time to talk with me today. My name is Qi Chen. I am working on my dissertation project and the goal of the study is to better understand how young adult cancer survivors’ experiences in using social media and what the perceived risk and benefits are. I am interested in any experiences and thoughts you have regarding your social media use before, during and after treatment with different platforms. I want to better understand young adult cancer survivors’ survivorship needs and social media use behaviors to figure out types of platforms and interventions that could help improve mental health outcomes on social media.

We have a few different topics to ask you about today. At any point you can stop me. Anything you share will be kept confidential and never linked with your identity. Because this is an exempted study from UT Austin IRB, which mean there is minimum risk participate in this study, we don’t require you to sign a consent form, but we will need your verbal consent to participate in the study and being recorded. I will be the only person having access to the video recording. The recording is only for the purpose of me transcribing the content. It will be destroyed after completing the study. Now, could you say your full name and whether we have your consent to participate in the interview and video record it?

Thank you, and before we start recording, do you have any questions for me or for the study?

First, I have some demographic questions for you:

1. How old are you? __________
2. What is your gender?

- Male
- Female
- Other

1. Do you consider yourself to be Hispanic or Latino?

- Yes, Hispanic or Latino
- No, Not Hispanic or Latino
- Prefer not to answer

1. What race do you consider yourself to be?
   (Please select all that apply)

- American Indian or Alaska Native
- Asian
- Black or African American
- Native Hawaiian or Other Pacific Islander
- White
- Other: ____________________
- Prefer not to answer

1. Are you currently enrolled in any kind of school (including undergraduate college or university, or graduate or professional school)?

- Yes
- No

If YES, what year are you?

- - 1st year undergraduate
  - 2nd year undergraduate
  - 3rd year undergraduate
  - 4th year undergraduate
  - 5th year undergraduate
  - Graduate or professional school

If NO, what is the highest degree or level of school you have completed?

- - Less than high school
  - High school degree
  - Some college or technical schooling
  - Two-year college degree
  - Four-year college degree
  - More than four-year college degree (e.g., graduate degree)

1. In what state do you currently live?

___________________________

1. What best describes the locality or environment in which you live?

- Urban
- Suburban
- Rural
- Prefer not to answer

1. Which of the following best describes your current employment status?

- I am employed full-time
- I am employed part-time
- I am not employed
- Prefer not to answer

1. What was your primary cancer diagnosis and stage?

- Brain tumor
- Breast
- Cervical
- Colon
- Hodgkin Lymphoma
- Leukemia
- Lung
- No-Hodgkin Lymphoma
- Ovarian
- Rectal
- Sarcoma
- Uterine/Endometrial
- Other:________________________________
- Prefer not to answer

1. How old were you when you started cancer treatment? ________ years
2. Which of the following best describes your current treatment status?

- In treatment
- Completed treatment
- On-going therapies (hormonal, immunotherapy, etc.)
- Chronic disease (in/out of treatment)
- Not yet started treatment
- Prefer not to answer

**Social media platforms usage**

Next, I would like to learn about social media you use and your experiences using them in treatment and after completion.

1. What are some of the social media apps or platforms that you currently use or have used?

For each of the platform:

- - What do you use it for? (Social, health information, entertainment, etc.)
  - What drew you to use this [said app/platforms]?
  - How often do you use and how much time do you spend on it each time you used?

1. What is your role on the platform? (Viewer, respondent, contributor, service user….) Probes:
   - - 1. What do you like/don’t like about viewing contents?
       2. what motivates you to share/interact with others online?
       3. What encourages/discourages you to use the online support group?
       4. What discourages you to share/interact with others?
       5. What do you view/respond/post/use?
2. Has your role on the social media changed during your cancer trajectory? How did it change and why?

**Social Media and Social Support**

1. How do you feel about the social support that you have received in real life (Tangible, emotional, informational, from family, friend, doctor)? Does it meet your need?
2. Have you used social media to look for health/cancer related information?

-If yes:

- - 1. What does the experience look like?

Probes:

- what do you like about using social media to find cancer information?
- What are things you don’t like about?
- Any negative/frustrated experience?
- Have you experienced any obstacles finding or understanding information?
  - 1. Do you trust the information you find? Why?

-If no: why?

1. Have you used social media to find peer survivors?

-If yes:

1. What does the experience look like?

Probes:

- How do you find? Is it easy or difficult to find someone share similar experiences with you?
- How do you like your relationship with peer online?
- Does the relationship stay online, or you meet offline?
- Any negative/frustrated experience?

Why Instagram group chat international

Charlesy

Online support group

-If no:

1) Why? (Difficult to find or not interested?)

2) Is that something you are willing to try in the future?

1. Do you use social media to interact/communicate with your family/friend about cancer? (do you follow them or vice versa)

-If yes:

- 1. What do you like about using social media to communicate compared to face to face and what do you don’t like about?
  2. How do you think it change the relationship (become closer, distant, or stay the same)?
  3. Does it change throughout the cancer trajectory?

-If no:

1) Why?

1. Does COVID-19 affect the way/purpose you use social media? Is it temporarily or permanently

Probes:

-If yes: What’s the difference between pre- and post- COVID? (eg. frequency, purpose, ways of interaction)

-If no: Why

**Closing**

1. What are things that you find most helpful on social media?
2. How much do you think social media change your cancer experience and in what aspects?
3. What do you like and don’t like about the current social media options in terms of meeting your needs?
4. Is there anything else you think it is important that relate to your social media use that I didn’t ask you today?
